# Biliary Anatomy Visualization and Surgeon Satisfaction Using Standard Cholangiography versus Indocyanine Green Fluorescent Cholangiography during Elective Laparoscopic Cholecystectomy: A Randomized Controlled Trial

**DOI:** 10.3390/jcm13030864

**Published:** 2024-02-01

**Authors:** Savvas Symeonidis, Ioannis Mantzoros, Elissavet Anestiadou, Orestis Ioannidis, Panagiotis Christidis, Stefanos Bitsianis, Konstantinos Zapsalis, Trigona Karastergiou, Dimitra Athanasiou, Stylianos Apostolidis, Stamatios Angelopoulos

**Affiliations:** 1 4th Department of General Surgery, Aristotle University of Thessaloniki, General Hospital of Thessaloniki “G. Papanikolaou”, 54124 Thessaloniki, Greece; simeonidissavvas@yahoo.com (S.S.); imanvol@gmail.com (I.M.); elissavetxatz@gmail.com (E.A.); panagiotischristidis13@gmail.com (P.C.); sbitsiani@gmail.com (S.B.); konstzapsalis@yahoo.gr (K.Z.); n.karastergiou@gmail.com (T.K.); dimitra071990@hotmail.gr (D.A.); saggelopoulos@auth.gr (S.A.); 2 1st Propaedeutic Department of Surgery, AHEPA University Hospital, Aristotle University of Thessaloniki, 54636 Thessaloniki, Greece; stlsa@med.auth.gr

**Keywords:** indocyanine green (ICG), near-infrared fluorescent cholangiography, elective laparoscopic cholecystectomy, bile duct injury, biliary mapping, intraoperative cholangiography

## Abstract

**Background**: Intraoperative biliary anatomy recognition is crucial for safety during laparoscopic cholecystectomy, since iatrogenic bile duct injuries represent a fatal complication, occurring in up to 0.9% of patients. Indocyanine green fluorescence cholangiography (ICG-FC) is a safe and cost-effective procedure for achieving a critical view of safety and recognizing early biliary injuries. The aim of this study is to compare the perioperative outcomes, usefulness and safety of standard intraoperative cholangiography (IOC) with ICG-FC with intravenous ICG. **Methods**: Between 1 June 2021 and 31 December 2022, 160 patients undergoing elective LC were randomized into two equal groups: Group A (standard IOC) and group B (ICG-FC with intravenous ICG). **Results**: No significant difference was found between the two groups regarding demographics, surgery indication or surgery duration. No significant difference was found regarding the visualization of critical biliary structures. However, the surgeon satisfaction and cholangiography duration presented significant differences in favor of ICG-FC. Regarding the inflammatory response, a significant difference between the two groups was found only in postoperative WBC levels. Hepatic and renal function test results were not significantly different between the two groups on the first postoperative day, except for direct bilirubin. No statistically significant difference was noted regarding 30-day postoperative complications, while none of the complications noted included bile duct injury events. **Conclusions**: ICG-FC presents equivalent results to IOC regarding extrahepatic biliary visualization and postoperative complications. However, more studies need to be performed in order to standardize the optimal dose, timing and mode of administration.

## 1. Introduction

Cholelithiasis affects up to 20% of the population in Western countries, and more than 750,000 cholecystectomies, mainly laparoscopic, are performed annually in the United States of America, rendering it the most common elective abdominal surgical procedure [1]. Laparoscopic cholecystectomy is considered a safe procedure, with minimum morbidity (1.6–5.3%) and mortality (0.08–0.14%) rates, while conversion rates to open cholecystectomy range between 4.2% and 6.2% [2]. However, laparoscopic cholecystectomy has been associated with higher rates of bile duct injuries (BDIs), up to 0.6%, when compared to open cholecystectomy (0.2–0.3% of cases). Despite the decreasing rate of BDIs nowadays compared to the early era of the laparoscopic cholecystectomy, the BDIs that currently occur are characterized by increased severity [3]. Apart from effects on morbidity and mortality, BDIs are accompanied by a significant long-term decrease in quality of life, both physical and mental, even after successful reconstruction surgery [4]. It is stated that in 71–97% of BDIs, the underlying cause is the misinterpretation of biliary anatomy [5]. Way et al. suggested that in 97% of cases, BDIs are result of a “visual perceptual illusion” due to an erroneous interpretation of local anatomy [6]. It is reported that 34% to 49% of surgeons will face a BDI in their career [7]. An important measure for the prevention of biliary and vascular injuries due to anatomical variations or intense inflammation is the achievement of a “critical view of safety” (CVS), as proposed by Strasberg et al. [8].

Yet, to date, there have been numerous cases in which application of the CVS intraoperatively is not enough for safe structure recognition [9]. Intraoperative mapping and visualization of bile duct anatomy as well as anatomical variants hold a key role in the attempt to eliminate or recognize early iatrogenic BDIs. Numerous cholangiography techniques have been proposed to ensure the correct interpretation of biliary structures, decreasing the incidence of BDIs to around 0.23% and 0.30% [10]. These methods include intraoperative cholangiography (IOC), dye cholangiography, light cholangiography, passive infrared cholangiography, near-infrared fluorescence cholangiography, hyperspectral cholangiography and laparoscopic ultrasound (LUS) [11].

Traditionally, IOC was used to detect choledocholithiasis and to prevent or diagnose bile duct injuries during the dissection of the hepatocystic triangle by delineating biliary tree anatomy and identifying abnormalities, such as fistulas or lesions [12]. However, the emersion of non-invasive imaging modalities, such as magnetic resonance cholangiopancreatography (MRCP), and the minimally invasive character of endoscopic retrograde cholangiopancreatography (ERCP) have rendered IOC as an optional tool during laparoscopic cholecystectomy by many surgeons [13]. In addition, IOC is associated with some disadvantages, including the need for additional staff and equipment, irradiation exposure, possible misinterpretation of the cholangiogram and the need for cystic duct cannulation [14]. Moreover, IOC is associated with a longer operative time; thus, one could assume that it leads to increased surgical stress and inflammatory responses. However, such an association has not been investigated yet. The most important weakness of IOC, however, is the need for dissection prior to visualization, which is technically challenging in the absence of anatomic landmarks, such as in inflammatory disease [15]. In conclusion, routine IOC performance may contribute to the early identification of a BDI, but it does not always prevent BDI, while injections of contrast material into the biliary tree may increase the risk of BDIs [16].

Ishizawa et al. first reported use of indocyanine green (ICG) for biliary tree mapping in 13 patients who underwent a hepatectomy and in 10 patients who underwent an open cholecystectomy in 2009 [17] ICG is a fluorescent tricarbocyanine dye that is activated with NIR, with absorption and emission wave peaks at 805–835 nm, respectively. When administered intravenously, ICG binds to plasma proteins and is excreted to the bile, enabling the identification of biliary structures with specific cameras [18]. ICG administration presents an excellent safety profile for human use, with contraindication only for patients with a known iodine allergy [19]. ICG fluorescence cholangiography (ICG-FC) is a noninvasive method for the real-time, intraoperative assessment of extrahepatic biliary anatomy, offering visualization even before the dissection of Calot’s triangle [20]. In addition, during ICG-FC, there is no need for cystic duct incision or irradiation exposure [21]. Numerous studies support the superiority of ICG-FC compared to standard white light for bile duct anatomy visualization [22]. A systematic review by Vlek et al. reports moderate-quality evidence for the comparison of ICG-FC to standard IOC [21]. However, further studies for the evaluation of ICG-FC’s efficacy under a variety of acute and chronic gallbladder inflammatory conditions, as well as for the establishment of timing and the dose of administration, are needed. 

Herein, in our single-center RCT study, we compared the efficacy and the peri- and postoperative results of ICG-FC and IOC for biliary recognition during elective laparoscopic cholecystectomy.

## 2. Materials and Methods

### 2.1. Study Design

This study is a non-inferiority, single-blind, randomized, controlled trial including 160 patients undergoing an elective laparoscopic cholecystectomy with either X-ray cholangiography (group A) or intraoperative fluorescence cholangiography (group B) for cholelithiasis or uncomplicated gallstone disease in a tertiary training hospital of large surgical volume in Greece, in the period between 1 June 2021 and 31 December 2022. Inclusion criteria were the following: (1) age > 18 years old, (2) written consent after extended oral and written information preoperatively, and (3) cholelithiasis or uncomplicated gallstone disease treated with elective laparoscopic cholecystectomy. The exclusion criteria included a (1) history of allergic reaction to iodine products, (2) pregnancy or lactation, (3) open cholecystectomy, (4) thyroid disorders, (5) an inability or unwillingness to give written consent or withdrawal of consent for any reason, (6) urgent or emergent cholecystectomy, (7) suspicion of malignant disease of the gallbladder, and (8) hepatic and renal failure. In suspicion of choledocholithiasis or cholangitis, patients underwent an ultrasound and MRCP prior to surgery, and if positive, preoperative ERCP was performed. This study was conducted in accordance with the Helsinki Declaration of 1975 [23]. This study is registered with ClinicalTrials.gov (NCT04908826).

We collected data, including patient demographics and biometric data (age, sex, BMI); previous medical history; the indication of surgery and operative time; biliary structure visualization; levels of inflammatory response indexes; preoperative and 1st postoperative values of kidney and liver function panel tests; basic coagulation tests; the duration of cholangiography; intraoperative and 1st-postoperative-day complications; and surgeon satisfaction. 

All procedures were performed by a team of three experienced consultant laparoscopic surgeons, with respect to the critical view of safety technique and to consensus conference-based guidelines [24]. All patients participating were administered antibiotics preoperatively and were attended for a 6-month follow-up period.

The operation-room personnel had a deep knowledge of the equipment needed and techniques performed. Non-eligible patients were also recorded for a later drop-out analysis.

### 2.2. Randomization and Blinding Process

Eligible patients, 160 in total, were included in the study and randomized to group A or group B with the use of a web-based password-protected database (SecuTrial, v. 5.6.2.2, interActive Systems GmbH, Berlin, Germany) at a 1:1 allocation ratio. The randomization process was performed after enrollment, and authors S.S. and O.I. were responsible for the written consent obtained from the patients. The patients were blinded regarding randomization. Patient data were anonymized during recording and analysis. 

### 2.3. Surgical Technique

In both groups, the operators followed the standard surgical technique (“American technique”), with the patient in the supine position and placement of four trocars. The surgeon and the assistant were standing on the left side of the patient [25].

#### 2.3.1. Intraoperative X-ray Cholangiography (IOC)

After the dissection of Calot’s triangle and the identification of the cystic duct and cystic artery, a titanium clip was placed close to the junction of the cystic duct and the infundibulum. A hemicircumferential incision was made on the cystic duct, just distal to the clip, using laparoscopic scissors and selective catheterization of the cystic duct, followed by the use of a cholangiography catheter of a 5Fr diameter. A grasper was also used to facilitate cannulation. The permeability of the catheter and leakage were checked by flushing 3 mL of normal saline (0.9%) into the lumen. A C-arm machine (Ziehm Imaging, Nürnberg, Germany) was placed in order to focus on the right upper quadrant, and 10 mL of Xenetix (iobitriol) diluted in 10 mL of normal Saline (0.9%) was administered through the catheter until the common bile duct was completely visualized. The cholangiography duration was measured as the time from the application of the catheter until its removal after obtaining satisfactory dynamic images.

#### 2.3.2. Intraoperative Fluorescence Cholangiography

Under sterile conditions, one vial of VERDYE (Diagnostic Green GmbH, Achheeim-Dornach, Germany), containing, 25 mg of indocyanine green, was reconstituted in 10 mL of sterile water for injection, and gentle shaking of the solution followed, according to the manufacturer’s guidelines. This procedure led to a solution with a concentration of 2.5 mg/mL. Six hours before anesthesia induction, 0.3 mg/kg of ICG was administered intravenously via a central or peripheral venous line, immediately followed by a 10 mL bolus of saline. The laparoscopic equipment, a KARL STORZ (Tuttlingen, Germany), including an HD camera IMAGE 1 S, with an easily switchable white-light–near-infrared fluorescence mode, was used. 

After trocar placement and an inspection of the peritoneal cavity, the operative field was inspected both under white light and in the ICG fluorescence mode before the dissection of Calot’s triangle in order to identify a safe landmark to begin the dissection. In addition, the ICG fluorescence mode was used for visualizing the main extrahepatic biliary structures, as well as for achieving a critical view of safety. The duration of the fluorescence cholangiography procedure was defined as the total time in ICG fluorescence mode.

### 2.4. Statistical Analysis

The measured variables were checked for the normality of their distribution by the Shapiro–Wilk test. Normally distributed, continuous variables were expressed by the arithmetic mean ± standard deviation (mean ± SD), while continuous variables with non-parametric distribution were expressed by the median and intra-quadratic range (median, IQR). Qualitative variables, categorical or ordinal, are presented as numbers and percentages per 100. The confidence interval was set at 95%, which means that the differences between the groups were considered statistically significant when *p* < 0.05. To compare the independent continuous variables in the two study groups, a *t*-test was used. To compare the continuous, dependent variables in each study group (pre-operatively vs. postoperatively), a paired *t*-test was used. To compare two qualitative variables in the two independent study groups, chi-square (χ^2^) was used. To compare multiple qualitative variables in the two independent study groups, a chi-square (χ^2^) test of independence was used. Parametric tests were preferred due to the sample size (*n* > 50 in each study group). The statistical analysis of the results was performed using the statistical program Jamovi Version 1. 6. 18.0.

### 2.5. Outcomes

The primary outcome was the visualization rate of a series of crucial anatomical landmarks, including the cystohepatic junction (the critical junction between the cystic duct, common hepatic duct and common bile duct; its successful recognition requires visualization of at least 1 cm of each structure distal from the confluence), the common hepatic duct (from the cystohepatic junction to the bifurcation), the common bile duct (from the cystohepatic junction to the retroduodenal part) and the cystic duct (from the cystohepatic junction to the origin from the gallbladder). In addition, the primary outcomes included the levels of inflammatory response indexes, the duration of cholangiography and surgeon satisfaction. Surgeon satisfaction was recorded immediately after surgery using a 5-point Likert scale (5: excellent, 4: good, 3: satisfactory, 2: poor, 1: very poor) [26]. 

The secondary outcomes included intraoperative and 1st-postoperative-day complications, as well as the rate of failed cholangiography and preoperative and 1st postoperative values of kidney and liver function panel tests and basic coagulation tests.

## 3. Results

**Figure 1:**
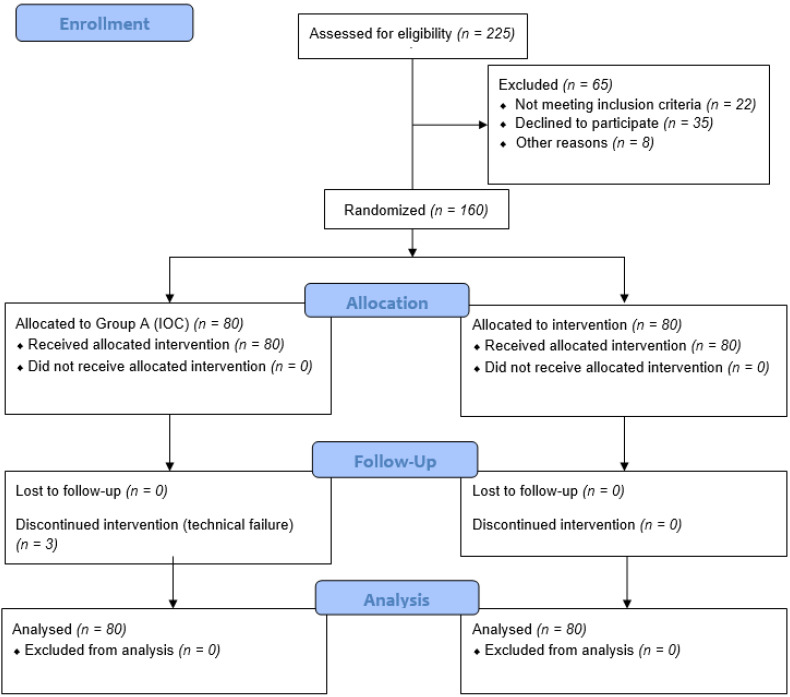
CONSORT 2010 diagram of the trial.

Of the 225 patients initially assessed for eligibility, 160 patients were finally included (Figure 1). The main exclusion reasons included no written informed consent, withdrawal of consent and allergy to iodine products. After randomization, 80 patients were allocated to each group and were included in final analysis.

No statistically significant differences were observed between the two groups regarding sex, age, BMI, ASA score or indication of surgery (Table 1). 

In group A, the attempt at X-ray cholangiography was unsuccessful in 3/80 patients due to obstruction of the cystic duct, while in group B, all patients (80/80) underwent ICG-fluorescence cholangiography. The operation was converted to open cholecystectomy in these three patients of group A due to an inability to perform IOC. 

There was no significant difference regarding the visualization of the critical junction (*p* = 0.827), common hepatic duct (*p* = 0.940), cystic duct (*p* = 0.225) and common bile duct (*p* = 0.276) between the two groups. The extrahepatic biliary anatomy is summarized in Table 2. However, a statistically significant difference between groups A and B was found regarding the cholangiography time (305 ± 18.6 vs. 110 ± 11.1 s, respectively, *p* < 0.001), while this difference was not reflected in the total median operating time, including both procedures of cholecystectomy and cholangiography for groups A vs. B (47.1 ± 7.31 min vs. 46.5 ± 7.43, *p* = 0.858). Regarding the surgeon assessment of the ease and feasibility of intraoperative cholangiography, expressed in an arbitrarily chosen numerical rating scale of one to five, a statistically significant intergroup difference was found in favor of ICG cholangiography: 2.9 ± 0.79 vs. 4.1 ± 1.05 for groups A and B, respectively (*p* < 0.001).

The differences between the two groups regarding the first-postoperative-day values of inflammatory response indexes (WBC, CRP, IL-6 and TNF-a) are presented in Table 3. A statistically significant difference between the two groups was found only in the postoperative WBC levels (9.02 ± 2.20 vs. 7.33 ± 1.13, *p* = 0.045) for groups A and B, respectively (*p* < 0.045). The hepatic function was not significantly different between the two groups on the first postoperative day, as reflected by the liver function test and coagulopathy profile, except for the levels of direct bilirubin (0.320 ± 0.132 vs. 0.154 ± 0.0381, *p* = 0.001). Finally, the renal function panel tests were not significantly different between the two groups on the first postoperative day.

Regarding common bile duct stone identification, choledocholithiasis was identified intraoperatively in two patients in the IOC group and in one patient in the ICG-FC group. For patients in the IOC group, a conversion to open cholecystectomy and common bile duct exploration was decided due to an impaired visualization of the total of extrahepatic biliary tree. In the ICG-FC group, choledocholithiasis was hypothesized after extensive dissection of the hepatoduodenal ligament and was confirmed and managed with concomitant ERCP during surgery.

No statistically significant difference was noted regarding 30-day postoperative complications (5.0% vs. 3.75%, *p* = 0.305). None of these complications were relevant to bile duct injury events. Two patients in group A developed mild biliary pancreatitis (Clavien–Dindo II) on postoperative days 3 and 4, and two patients developed subhepatic hematoma on postoperative day 4, which was managed conservatively due to small size (Clavien–Dindo II). One patient from group B developed a paralytic postoperative ileus (Clavien–Dindo II) on postoperative day 2, while two patients presented with atelectasis and fever (Clavien–Dindo II) on postoperative day 2 due to poor mobilization and a history of COPD. No intraoperative complications were noted in neither of two groups. Intraoperatively, choledocholithiasis of the common bile duct was detected in two patients in group A and in one patient in group B. Both patients underwent postoperative ERCP and stone removal without complications.

All costs were calculated and presented in euros (EUR) and included only expendables, since our hospital was equipped with a C-arm machine and laparoscopic equipment, including a light box, a light cord, a camera with a white-light–near-infrared fluorescence mode, and a laparoscope. For ICG-FC, the cost includes the price of vial of VERDYE, based on the recommended dose of 0.3 mg/kg of the patient’s weight. ICG was calculated at a price of EUR 0.8825/kg of the patient’s weight, while the cost of a VERDYE vial is approximately EUR 155.91. Regarding IOC, the final cost included the cost of the cholangiography catheter, approximately EUR 10 per patient, and of the iodinated contrast, with a cost of EUR 10 per vial of 20mL.

## 4. Discussion

Laparoscopic cholecystectomy is the gold-standard treatment for benign gallbladder diseases and is among the most commonly performed procedures by general surgeons, [27], with more than 750,000 cholecystectomies performed annually [1]. The emersion of laparoscopic cholecystectomy has been associated with an increasing trend in BDI rates and severity, found to range up to 0.6% for laparoscopic versus 0.1% for open cholecystectomy [28]. Despite the relatively low rate of BDIs, the burden of this complication is rendered particularly important, if we consider that, in the USA, approximately 30 million adults suffer from gallstone disease, increasing the absolute number of noted BDIs. In addition, it is stated that the real incidence of BDIs is higher than the rate reported due to the absence of consensus in the definition of BDIs and data collection bias [29]. 

Numerous mechanisms have been proposed regarding the etiology of BDIs during LC, with misinterpretation of the biliary anatomy and technical pitfalls being the most prevalent. In particular, in 70% of bile duct injuries, misinterpretation of the common bile duct for the cystic duct or of an aberrant right hepatic duct as the cystic duct can be accused [30]. Numerous attempts have been made to reduce the rate of BDIs [31]. Among the variety of techniques proposed for biliary anatomy mapping and reducing BDIs, the most commonly used are IOC, intraoperative ultrasound, obtaining the critical view of safety and NIR-ICG fluorescence cholangiography. 

Initially proposed by Mirizzi et al. in 1931, IOC remains the gold-standard technique for the intraoperative visualization of extrahepatic biliary anatomy during LC [32]. The procedure involves the dissection of Calot’s triangle, the cannulation of the cystic duct and the injection of a contrast dye into it. The flow of the contrast agent through the biliary tree permits a dynamic, real-time visualization of the biliary anatomy [33]. However, IOC use in clinical practice is limited and is selectively performed based on surgeon preference [34]. Routine performance of IOC has achieved an increase in intraoperative detection of BDIs from 45% to 85%, according to Archer et al., reducing the associated morbidity and length of hospitalization [35]. In addition, IOC contributes to delineating challenging biliary anatomy and the detection of choledocholithiasis [31]. On the contrary, IOC is associated with a series of technical difficulties and weaknesses, including a significant increase in operating time, additional costs due to radiological equipment and personnel, additional risks of BDI during cystic duct cannulation and radiation exposure [20]. Furthermore, IOC is accompanied by a failure rate of approximately 15% due to fibrotic and inflammatory tissue in the triangle of Calot, obstruction of the cystic duct or difficulty in the dissection and catheterization of the cystic duct. Finally, a high rate of false-positive results, ranging from 2 to 16%, are associated with IOC, incorrectly suggesting the presence of CBD stones [36]. 

ICG-FC is an emerging and evolving technique in the field of extrahepatic biliary mapping during laparoscopic cholecystectomy. Its mechanism of action is based on the selective metabolism and exclusive secretion of ICG into the bile and the light emission from protein-bound ICG under near-infrared light [37]. ICG reaches its peak concentration in the bile between 30 and 120 min after administration, while peak concentration in the blood is noticed within 1–2 min [38]. ICG-FC is a safe method of real-time biliary visualization, which does not necessitate radiation exposure and does not alter surgery duration, nor does it need a special learning curve [39]. ICG fluorescence presents a series of advantages. It is a non-invasive modality, which does not require manipulation of the cystic duct, thus limiting the rate of iatrogenic BDI. The ability for an easy and fast switch between the standard white-light mode and NIR fluorescence enables sufficient real-time biliary mapping, while near-infrared fluorescence cholangiography does not require a patent cystic duct and constitutes a valuable tool for the education of novice surgeons, increasing intraoperative safety [40]. In the case–control study by Quaresima et al., the authors concluded that ICG-FC led to an increased number of cholecystectomies, even in challenging cases of acute cholecystitis and empyema, performed by resident doctors due to increased intraoperative confidence in delineating anatomical landmarks [41]. Despite the promising observation of Quaresima et al., all of our patients from both groups underwent laparoscopic cholecystectomy performed by a consultant general surgeon, with experience in laparoscopic surgery, to avoid bias. 

No consensus has been reached in the literature regarding the optimal dosage and time of ICG injection [42], with most authors suggesting a dose of 2.5 mg administered 2–6 h preoperatively [43,44,45]. One important weakness of this technique is the high noise-to-signal ratio produced by the hepatic parenchyma, which is strong enough to interfere with biliary structures. Liver enhancement may reduce the clarity of the biliary anatomy due to prolonged ICG fluorescence in humans [44]. Despite the fact that liver enhancement was not directly evaluated in our study, the surgeon satisfaction scores from ICG-FC indicate that it was not important enough to impair biliary recognition under NIR light. One should also take into consideration that conditions such as hepatic failure or biliary excretion problems due to limited flow to the common bile duct may alter or limit ICG fluorescence [46]. Similarly, the visualization value of ICG-FC may be restricted by excessive adipose tissue or extensive fibrotic tissue, such as in patients with a high BMI or with a history of multiple episodes of inflammation due to an inability to penetrate tissues thicker than 10 mm after the intravenous administration of ICG [47]. In addition, despite the fact that intravenous ICG administration is most commonly described, the literature contains a series of reports suggesting direct ICG injection into the gallbladder in order to achieve the lower dose needed and to eliminate background liver enhancement [10,48,49]. 

Our study showed that ICG-FC was not inferior to IOC in terms of extrahepatic biliary tree visualization. More specifically, no statistically significant difference was found between the two groups regarding the visualization of the critical junction of the common bile duct–cystic duct, as well as the visualization of the common hepatic duct, cystic duct and common bile duct. These findings prove the non-inferiority of ICG-FC, compared to IOC, in terms of the intraoperative visualization of the biliary tract, and are in accordance with the results of systematic reviews comparing the two methods. Vlek et al., in their systematic review including nineteen studies, concluded that, despite the fact that the CD was more frequently visualized with ICG-FC than IOC, similarly to our results, this difference was not statistically significant. Equal results were also encountered for the other important structures of the biliary tract, rendering ICG-FC a comparable method to conventional IOC [21]. In contrary to the aforementioned results, Lim et al. reported higher CD, CBD and CD-CBD junction visualization rates by ICG-FC, compared to IOC, yet without a proven statistically significant difference. In contrary, a statistically significant difference was achieved regarding higher CHD visualization rates using ICG-FC compared to IOC [50]. The aforementioned modalities have also successfully been used for biliary visualization during robotic cholecystectomy in a case series of 58 patients, with reported rates of cystic–common bile duct junction recognition of approximately 98.15% and 96.15% for ICG-FC and IOC, respectively [51].

Regarding cholangiography duration, the average time spent on ICG-FC was 110 ± 11.1 s, which is significantly lower, compared to 305 ± 18.6 s spent on IOC (*p* < 0.001). This finding is in accordance with reports in the literature [52]. However, regarding the total operative time, no statistically significant difference was noted between the two groups, which comes in contrary with the results of the literature, suggesting a shorter operative duration in patients undergoing ICG-FC due to the easier and earlier recognition of anatomy and the longer time needed to perform IOC [41]. Cystic duct impaction led to the failure of cholangiography in three patients of the IOC group, while in the ICF-FC group, cholangiography was achieved in all cases. This is an important advantage of ICG-FC: visualization of the biliary tree, even in cases of acute cholecystitis with a non-patent cystic duct [52]. Indeed, in the ICG-FC group, fluorescent cholangiography was achieved in 100% of cases, which is in accordance with the rate reported in the literature [41].

The performance of biochemical liver function tests (LFTs), including alanine aminotransferase (ALT), aspartate aminotransferase (AST), gamma-glutamyl transpeptidase (GGT), alkaline phosphatase (ALP) and bilirubin, both direct and indirect, is one of the most common methods for the assessment of biliary injuries or duct obstruction postoperatively [53]. In our study, the aforementioned tests were performed, revealing a statistically significant difference only in the values of the first-postoperative-day direct bilirubin between the IOC and ICG-FC groups, with higher values encountered in the first group. 

CRP is a common acute-phase biomarker used to investigate postoperative inflammatory responses, but it is also a tentative stress marker for quantifying perioperative immune modulation [54]. In addition, a series of other inflammatory cytokines, such as IL-6 and TNF-α, are responsible for promoting the systemic changes known as the acute-phase response following surgical trauma [55]. It has been proven that laparoscopic cholecystectomy leads to a reduced extent of surgical stress and inflammatory response compared to open cholecystectomy, with this difference also being reflected in terms of postoperative morbidity and the length and cost of the hospital stay [56]. In addition, alteration in the pro-inflammatory response in laparoscopic cholecystectomy, when compared to open cholecystectomy, has led to alterations in postoperative pain [57]. However, the BDI rate with laparoscopic cholecystectomy is nearly triple compared to open cholecystectomy, constituting a weakness of this minimally invasive procedure [58]. Haalem et al., in their prospective study, comparing surgical stress induced by laparoscopic and open cholecystectomy, concluded that there was no statistical difference in the levels of CRP and TNF-α, attributed to the limited tissue trauma caused by both methods. However, this study excluded patients who required perioperative cholangiography [59]. The literature has no evidence regarding the effect of BDI prevention and detection techniques, such as IOC and ICG-FC, on surgical stress and the inflammatory cascade. To the best of our knowledge, the present study is the first to measure levels of inflammatory biomarkers after laparoscopic cholecystectomy in an effort to quantify the additional surgical stress triggered by biliary mapping procedures. Among the biomarkers investigated for the evaluation of surgical stress, only the WBC levels on the first postoperative day presented a significant difference between the two groups, with higher levels in the IOC group. However, the mean values of both groups were within the normal range, thus suggesting that neither of the methods significantly burden the patient intraoperatively. 

The literature includes numerous studies investigating the role of laparoscopic cholecystectomy in liver function, since, apart from surgical dissection and the manipulation of crucial structures, factors such as impaired portal vein flow, decreased venous flow and variations in intraperitoneal pressure during laparoscopy may alter hepatic function [60]. More specifically, the impairment of blood flow to the hepatic parenchyma may obstruct the production of proteins derived from hepatocytes, such as PT, PTT and INR. Two independent studies published by Shahraki et al. and Garg et al. revealed a significant change only in PT after laparoscopic cholecystectomy, with no significant effects on other coagulation factors. This difference was attributed to the severe catabolic response of the liver postoperatively [61,62]. Regarding the effect of intraoperative biliary mapping on the coagulation mechanism, the literature evidence is scarce. In our study, no significant differences emerged postoperatively between the methods of biliary visualization, suggesting that neither IOC nor ICG-FC led to a significant impairment of hepatic function. Similarly, the effects of laparoscopic cholecystectomy on renal function were studied by Bansal et la, who concluded that no significant differences presented in the renal function profile before and after laparoscopic cholecystectomy [63]. In our study, we focused on the changes in renal function tests after IOC and ICG-FC during laparoscopic cholecystectomy. None of the two methods affected the first-postoperative-day levels of urea and creatinine in a significant way, proving that both techniques can be safely applied without the risk of renal impairment. However, it should be highlighted that the present study excluded patients with renal failure, so conclusions should be drawn cautiously regarding this special group of patients. However, in a clinical audit published in 2000, Pietra et al. reported the performance of IOC in patients at risk of contrast-associated acute renal failure, instead of intravenous cholangiography [64].

The surgeon satisfaction level has been widely associated with technical challenges and intraoperative complications [65]. The techniques of intraoperative biliary mapping are an useful tool, providing guidance and a reduced complication rate. Regarding the surgeon-reported ease of cholangiography performance, Lehrskov et al. used a numerical rating scale from one to five, with a statistically significant difference in favor of ICG-FC compared to IOC [52]. Quaresima et al., also using a visual analog scale from one to five, with five being the most accurate image acquired, concluded a visualization score of ≥3 in 41 of 44 patients after the dissection of Calot’s triangle, while no data are reported regarding the IOC group [41]. On the contrary, the quality of the intraoperative visualization of the biliary anatomy was also evaluated in the study of Diana et al. using a five-point Likert scale. The results revealed the significant superiority of IOC-acquired images compared to ICG-FC images [51]. Our results show significantly higher levels of surgeon satisfaction during ICG-FC, rendering it a feasible and easy-to-use mapping modality. 

Studies on cost-effectiveness comparing the two abovementioned methods of biliary visualization are, in general, scarce, while numerous studies have been performed for each technique separately [66,67]. Dip and colleagues performed a cost analysis and effectiveness analysis. In general, the market value of the mobile fluoroscopic image-intensifier system (C-arm machine) is approximately USD 75,000, while the total costs also included the cholangiogram catheter cost and radiology report. Regarding the costs of the equipment for indocyanine green fluorescent cholangiography, it has been calculated that the cost of necessary devices, including the light box, the light cord, a camera with an easily switchable white-light–near-infrared fluorescence mode, and a laparoscope, is increased by approximately 20% when compared with the standard conventional high-definition laparoscopic system. Finally, regarding the expendables’ cost, ICG dye costs approximately twice that of iodinated contrast, while the final cost of standard cholangiography was USD 778.83, compared to the cost of ICG fluorescent cholangiography, which was approximately USD 32.3 [68]. According to a cost-effectiveness analysis conducted by Reeves et, fluorescent cholangiography leads to reduced lifetime costs by USD 1235 per patient when compared to standard white-light laparoscopic cholecystectomy by decreasing the operative duration and rate of conversion to open cholecystectomy [69]. On the contrary, routine intraoperative cholangiography during laparoscopic cholecystectomy was accompanied with additional hospital costs of USD 706–739 [67]. However, further studies comparing the cost and efficacy of standard cholangiography versus indocyanine green fluorescent cholangiography are necessary in order to be able to draw safe conclusions.

This study also presents a series of limitations. First of all, the interest of visualization was focused on the biliary structures which are necessary to obtain a critical view of safety, and thus, visualization of the left and right hepatic ducts was not recorded. However, the literature includes reports of hepatic duct visualization with various cholangiography modalities. Lehrskov et al., in their RCT, reported a statistically significant difference in favor of IOC regarding the visualization of both hepatic ducts (16 in the ICG-FC group vs. 51 in the IOC group, *p* < 0.001). This significant difference was attributed to the low penetration depth of the emitted light through the overlying liver parenchyma and dense fibrotic and fatty tissue [52]. The limitation of penetration depth is among the most important weaknesses of ICG fluorescence, since the maximum penetration depth of NIRF visualization is 1.5 cm [70,71]. Similarly, a prospective cohort study by Dip et al., comparing the visualization of biliary structures in obese and non-obese patients under ICG fluorescence, concluded that there were no significant differences in hepatic duct, common bile duct and cystic duct visualization between obese and non-obese groups. However, obesity was found to a significant covariate in hepatic duct visualization due to increased concentrations of dense adipose tissue in the porta hepatis, impairing the emission of fluorescent light [72]. In our study, although the BMI did not differ significantly between the groups, no measurements of visceral tissue percentage were performed. Moreover, in our study, ICG dye was administered six hours before anesthesia induction, in a dose of 0.3 mg/kg. It could be assumed that prolonging the interval time between administration and fluorescence could have improved the visualization rate. Furthermore, the cost analysis of the two methods included only the costs of expendables, excluding the calculation of equipment cost. In this way, further studies, including a cost analysis and studies on the effectiveness of the two methods of cholangiography, are necessary. In addition, our study only included patients undergoing elective laparoscopic cholecystectomy; thus, it cannot provide any conclusions regarding the feasibility and safety of ICG-FC in urgent and emergency settings.

## 5. Conclusions

Our RCT study showed that ICG-FC was equally as sufficient as IOC in the visualization of structures that are vital for avoiding BDI, such as the critical junction, CHD, CBD and CD, during laparoscopic cholecystectomy. However, the greater surgeon satisfaction score for ICG-FC enlightens the promising abilities of ICG-FC. More trials are necessary, however, for assessing the role of ICG-FC in the emergency setting, as well as for improving its results in special patient groups, such as obese patients.

## Figures and Tables

**Table 1:** Basic characteristics of two groups.

Variables		Group A (IOC) *N*_1_ = 80	Group B (ICG-FC) *N*_2_ = 80	*p*-Value
Age (years), m ± sd		56.1 ± 12.9	49.9 ± 10.7	0.259
Sex, No%	Female	57/80 (71.25%)	48/80 (60.0%)	0.639
	Male	23/80 (28.75%)	32/80 (24.0%)	-
BMI (kg/m^2^), m ± sd		28.3 ± 5.82	27.9 ± 3.51	0.826
ASA Score, No%	II	56/80 (70.0%)	55/80 (68.75%)	0.117
	III	24/80 (30.0%)	25/80 (31.25%)	-
Indications, No%	Cholelithiasis	76/80 (95%)	77/80 (96.25%)	0.144
	Adenomyomatosis	3/80 (3.75%)	1/80 (1.25%)	-
	Polyps	1/80 (1.25%)	2/80 (2.50%)	-
Duration of surgical procedure (min), m ± sd		47.1 ± 7.31	46.5 ± 7.43	0.858
Complications, No%	Yes	4/80 (5.0%)	3/80 (3.75%)	0.305
	No	76/80 (95.0%)	77/80 (96.25%)	-
Choledocholithiasis, No%	Yes	2/80 (2.50%)	1/80 (1.25%)	1.000
	No	78/80 (97.50%)	79/80 (98.75%)	-

ΙOC: Standard intraoperative cholangiography; ICG-FC: Indocyanine green fluorescence cholangiography; m: mean; sd: standard deviation; Nο: number; ASA: American American Society of Anesthesiologists.

**Table 2:** Extrahepatic bile duct anatomy visualization, duration of cholangiography and surgeon satisfaction among two groups.

Variables		Group A (IOC) *N*_1_ = 80	Group B (ICG-FC) *N*_2_ = 80	*p*-Value
No. of failed investigations		3/80 (3.75%)	0/80 (0.0%)	
Cholangiography time (sec), m ± sd		305 ± 18.6	110 ± 11.1	**< 0.001**
Critical junction visualization	Yes	71/77 (92.2%)	73/80 (91.25%)	0.827
	No	6/77 (7.8%)	7/80 (8.75%)	-
CHD visualization	Yes	70/77 (90.9%)	73/80 (91.25%)	0.940
	No	7/77 (9.1%)	7/80 (8.75%)	-
CBD visualization	Yes	71/77 (92.2%)	77/80 (96.25%)	0.276
	No	6/77 (7.8%)	3/80 (3.75%)	-
CD visualization	Yes	72/77 (93.5%)	78/80 (97.5%)	0.225
	No	5/77 (6.5%)	2/80 (2.5%)	-
Surgeon satisfaction, m ± sd		2.9 ± 0.79	4.1 ± 1.05	**<0.001**

ΙOC: Standard intraoperative cholangiography; ICG-FC: Indocyanine green fluorescence cholangiography; m: mean; sd: standard deviation; sec: seconds; No: number; CHD: common hepatic duct; CBD: common bile duct; CD: cystic duct.

**Table 3:** Results of laboratory tests among two groups in 1st postoperative day.

Variables	Group A (IOC) *N*_1_ = 80	Group B (ICG-FC) *N*_2_ = 80	*p*-Value
SGOT pre (g/dL), m ± sd	18.2 ± 3.82	20.7 ± 4.03	0.172
SGOT post (g/dL), m ± sd	60.1 ± 15.5	60.1 ± 11.5	1.000
SGPT pre (g/dL), m ± sd	18.2 ± 3.39	22.1 ± 3.41	**0.020**
SGPT post (g/dL), m ± sd	66.2 ± 13.4	55.0 ± 15.2	0.097
ALP pre (g/dL), m ± sd	87.2 ± 11.3	77.3 ± 11.4	0.067
ALP post (g/dL), m ± sd	75.5 ± 12.3	65.0 ± 12.0	0.070
γ-GT pre (g/dL), m ± sd	14.2 ± 2.97	14.9 ± 2.60	0.582
γ-GT post (g/dL), m ± sd	10.0 ± 2.75	9.80 ± 2.04	0.856
Indirect bilirubin pre (mg/dL), m ± sd	0.435 ± 0.248	0.365 ± 0.0880	0.412
Indirect bilirubin post (mg/dL), post (mg/dL), m ± sd	0.556 ± 0.253	0.532 ± 0.0977	0.705
Direct bilirubin pre (mg/dL), m ± sd	0.227 ± 0.110	0.211 ± 0.0314	0.664
Direct bilirubin post (mg/dL), m ± sd	0.320 ± 0.132	0.154 ± 0.0381	**0.001**
Total bilirubin pre (mg/dL), m ± sd	0.686 ± 0.367	0.576 ± 0.111	0.377
Total bilirubin post (mg/dL), m ± sd	0.861 ± 0.287	0.686 ± 0.124	0.093
PT pre (seconds), m ± sd	10.9 ± 0.584	11.1 ± 0.751	0.454
PT post (seconds), m ± sd	11.1 ± 0.753	11.1 ± 0.643	0.950
aPTT pre (seconds), m ± sd	27.4 ± 4.04	25.9 ± 2.21	0.346
aPTT post (seconds), m ± sd	27.9 ± 3.82	26.8 ± 2.32	0.442
INR pre (number), m ± sd	0.902 ± 0.0527	0.908 ± 0.0368	0.771
INR post (number), m ± sd	0.943 ± 0.0544	0.921 ± 0.0404	0.318
Urea pre (mg/dL), m ± sd	32.2 ± 9.31	30.6 ± 6.43	0.660
Urea post (mg/dL), m ± sd	27.3 ± 9.31	34.1 ± 10.5	0.143
Creatinine pre (mg/dL), m ± sd	0.713 ± 0.117	0.843 ± 0.145	**0.040**
Creatinine post (mg/dL), m ± sd	0.758 ± 0.186	0.726 ± 0.179	0.700
WBC pre (mm^3^/L), m ± sd	5.92 ± 1.74	5.08 ± 0.942	0.194
WBC post (mm^3^/L), m ± sd	9.02 ± 2.20	7.33 ± 1.13	**0.045**
CRP pre (mg/dL), m ± sd	0.610 ± 0.292	0.670 ± 0.250	0.628
CRP post (mg/dL), m ± sd	3.20 ± 2.42	5.05 ± 1.83	0.070
TNF-a pre (pg/dL), m ± sd	1.99 ± 0.223	2.06 ± 0.276	0.541
TNF-a post (pg/dL), m ± sd	2.09 ± 0.195	2.16 ± 0.227	0.457
IL-6 pre (pg/dL), m ± sd	0.910 ± 0.0745	0.895 ± 0.0642	0.635
IL-6 post (pg/dL), m ± sd	0.949 ± 0.0863	0.924 ± 0.0648	0.473

ΙOC: Standard intraoperative cholangiography; ICG-FC: Indocyanine green fluorescence cholangiography; m: mean; sd: standard deviation; L: liter; dL: deciliter; mg milligram; SGOT: serum glutamic-oxaloacetic transaminase; SGPT: serum glutamic pyruvic transaminase; ALP: alkaline phosphatase; γ-GT: gamma-glutamyl transferase; PT: prothrombin time; aPTT; activated partial thromboplastin time; INR: international normalised ratio; CRP: c-reactive protein; WBC: white blood cell; TNF: tumor necrosis factor; IL-6: interleukine-6.

## Data Availability

Data are available to any qualified researchers upon request to simeonidissavvas@yahoo.com.
